# 8-Anilino-1-naphthalenesulfonate-Conjugated Carbon-Coated Ferrite Nanodots for Fluoromagnetic Imaging, Smart Drug Delivery, and Biomolecular Sensing

**DOI:** 10.3390/pharmaceutics16111378

**Published:** 2024-10-26

**Authors:** Anbazhagan Thirumalai, Koyeli Girigoswami, Alex Daniel Prabhu, Pazhani Durgadevi, Venkatakrishnan Kiran, Agnishwar Girigoswami

**Affiliations:** 1 Medical Bionanotechnology, Faculty of Allied Health Sciences (FAHS), Chettinad Hospital & Research Institute (CHRI), Chettinad Academy of Research and Education (CARE), Chennai 603103, Tamil Nadu, India; athiru1999@gmail.com (A.T.); koyelig@care.edu.in (K.G.);; 2 Department of Radiology, Chettinad Hospital & Research Institute (CHRI), Chettinad Academy of Research and Education (CARE), Chennai 603103, Tamil Nadu, India; dralexdaniel@hotmail.com

**Keywords:** 8-anilino-1-naphthalenesulfonic acid, ANS, carbon dots, multimodal imaging, manganese ferrite, theranostic

## Abstract

Background: Superparamagnetic properties and excitation independence have been incorporated into carbon-decorated manganese ferrite nanodots (MnFe@C) to introduce an economical and safer multimodal agent for use in both T1-T2 MRI and fluorescence-based imaging to replace the conventional highly toxic heavy metal contrast agents. Methods: The surface conjugation of 8-anilino-1-naphthalenesulfonate (ANS) to MnFe@C nanodots (ANS-MnFe@C) enhances both longitudinal and transverse MRI relaxation, improves fluorescence for optical imaging, and increases protein detection sensitivity, showing higher multimodal efficacy in terms of molar relaxivity, radiant efficiencies, and fluorescence sensitivity compared to MnFe@C. Results: The band gap energy was determined using Tauc’s equation to be 3.32 eV, while a 72% quantum yield demonstrated that ANS-MnFe@C was highly fluorescent, with the linear range and association constant calculated using the Stern–Volmer relation. The synthesized ANS-MnFe@C demonstrated excellent selectivity and sensitivity for bovine serum albumin (BSA), with a nanomolar detection limit of 367.09 nM and a broad linear range from 0.015 to 0.225 mM. Conclusions: In conclusion, ANS-MnFe@C holds ease of fabrication, good biocompatibility, as assessed in A375 cells, and an effective pH-sensitive doxorubicin release profile to establish anticancer activity in lung cancer cell line (A549), highlighting its potential as an affordable therapeutic agent for multimodal imaging, drug delivery, and protein sensing.

## 1. Introduction

There are indeed various imaging methods and techniques being used for cellular abnormality detection, including magnetic resonance imaging (MRI), computed tomography (CT), X-ray, positron emission tomography (PET), fluorescence imaging, ultrasound, and single photon emission computed tomography (SPECT) [1,2,3]. MRI’s noninvasive approach and multidimensional tomography capabilities position it as a highly effective medical imaging technique. The use of advanced imaging agents can improve the contrast between various tissues or regions of interest, resulting in clearer and more detailed images [3,4]. MRI contrast agents typically work by altering the relaxation times of water proton signals and are classified into two types: longitudinal relaxation (T1) agents, which generate positive contrast images, and transverse relaxation (T2) agents, which produce negative contrast images [5]. Structural MRI is a commonly employed method for monitoring the effectiveness of therapeutic measures and diagnosing anatomical abnormalities, in that 1.5 Tesla or 3 Tesla strength of magnetic fields are commonly used for clinical imaging. Unlike traditional paramagnetic gadolinium, complex contrast agents such as DOTAREM, ProHance, Magnevist, and superparamagnetic nanoparticles are gaining prominence as the next-generation magnetic probes due to their outstanding magnetism and prolonged circulatory status [6].

In terms of the framework and utilization, iron oxide nanoparticles have made significant progress in a number of biomedicine applications; however, their innate negative contrast capabilities have limited their application in MR imaging. For T1 images, manganese chelates have emerged as a promising contrast agent [7,8,9]. Recent research shows that mixed ferrite nanoparticles, especially those doped with metals like MnFe_2_O_4_, have much higher magnetization than other similar nanoparticles. MnFe_2_O_4_ nanoparticles have been described as the strongest MR image contrast agent to date, with a much higher T2 relaxivity [10,11]. The biomedical application of manganese ions is hampered by notable challenges, including a decline in catalytic capabilities, compromised magnetic properties, and reduced dispersibility of susceptible magnetic nanoparticles during catalytic transformations [12,13,14]. Hence, a protective layer or shell is designed to enhance biocompatibility without compromising the magnetic and catalytic functions, thereby reducing uptake by the reticuloendothelial system (RES) [2,15,16]. Lee et al. designed nitrogen-containing graphene quantum dots doped with Mn^2+^ and Gd^3+^, which integrate both MRI and fluorescence for dual-modal biomedical uses [17]. These quantum dots are biocompatible, water-soluble, and efficiently absorbed by cells. They provide both T1 and T2 MRI contrast along with robust fluorescence, offering a safer and more scalable alternative to conventional MRI contrast agents [17]. Tiron et al. developed N-hydroxyphthalimide-derived carbon dots doped with gadolinium, Fe^3+^, and Mn^2+^ for theranostic applications, which combine anti-tumoral effects with MRI functionality [18]. The findings indicate that Mn^2+^-doped carbon dots hold the potential as effective theranostic agents for pre-clinical studies.

Creating a protective layer on magnetic nanoparticles can be accomplished by employing non-magnetic materials like carbon or silica, which will effectively address the challenges associated with compromised magnetic and catalytic functions characterized by a low refractive index and porous structure [19,20,21,22]. The chemical and mechanical stability of magnetic materials coated with carbon renders them resilient to variations in pH and temperature within the cellular microenvironment [23]. In addition to stabilizing the magnetic properties, coating carbon on the surface of magnetic particles develops outstanding optical properties and assists in the evolution of fluorescence-based imaging agents and biosensors [24]. Biocompatibility, photostability, scalability, and tunable excitation/emission wavelengths have made carbon dots (CDs) an attractive fluorescent material for biomedical applications [25,26]. CDs are 2D carbonaceous nanoparticles composed of cores characterized by sp^2^-conjugated carbon surrounded by amorphous shells containing carboxyl, amine, hydroxyl, and carbonyl groups [27]. Carbon nanomaterials have unique optical properties due to their sp^2^ hybridized carbon atom. π-bonds are formed by the arrangement of carbon atoms in a hexagonal lattice and are responsible for the characteristic π-electron states [28,29]. The surface defect leads to visible fluorescence emission because of the generation of electron–hole pairs. When photons are absorbed, electrons in the π-electron state are excited to the π* state, creating an electron–hole pair, which is strongly localized to the π and π* electronic levels of sp^2^ sites [30]. The bandgap is influenced by both σ (valence band) and σ* (conduction band) states, which are related to sp^3^ hybridization in the matrix. The π and π* states lie between the bandgap formed by the σ and σ* states; this arrangement contributes to unique properties, making them highly interesting materials in various applications, including biomedicine, biosensors, and optoelectronic devices [31]. Through surface functionalization or passivation, surface defects can become more stable and produce higher radiant recombination of surface-bound electrons, as well as greater radiant fluorescence emissions [32].

8-Anilino-1-naphthalenesulfonate (ANS) contains a hydrophobic anilinonapthalene group and charged sulfonate group, making it an amphipathic molecule with light green fluorescent properties [33,34,35]. The fluorescence yield of ANS was weak in water but significantly enhanced in fluorescence after the ANS was dissolved in a less polar solvent and the hydrophobic region of membranes or proteins [36]. Utilizing conjugation between CDs and ANS represents an alternative method for improving the fluorescence properties, and the modification can be achieved through diverse chemical approaches. ANS contains an aromatic ring (aniline), and sulfonic acid groups can facilitate the conjugation of the molecule onto the surface of CDs [37]. The amino group of CDs can react with the sulfo group of ANS to form a covalent interaction, or they can still interact through non-covalent interactions like electrostatic interactions, π-π stacking, or hydrogen bonding [38]. This ANS conjugation will enhance the fluorescence properties, hydrophobicity, solubility, binding affinity, sensitivity, biocompatibility, stability, and shelf life of CDs [39,40]. In this study, ANS was chosen to conjugate on the surface of carbon-coated manganese ferrite nanodots (ANS-MnFe@C) and doxorubicin was loaded (ANS-MnFe@C-Dox) to increase the multimodal diagnostic efficacy and sensitivity for the use of theranostic agents. The optical properties, magnetic properties, release kinetics, and sensitive detection of BSA were examined; the biocompatibility of ANS-MnFe@C and the anticancer activity of ANS-MnFe@C-Dox were investigated using a skin cancer cell line (A375) and lung cancer (A549) cell line.

## 2. Materials and Methods

### 2.1. Materials

Manganese(II) chloride tetrahydrate (MnCl_2_·4H_2_O) was supplied by SRL Chemical, Mumbai, India. Iron(III) chloride hexahydrate (FeCl_3_·6H_2_O) and 8-anilino-1-napthalenesulfonate were obtained from Sigma-Aldrich, St. Louis, MO, USA. Citric acid (C_6_H_8_O_7_), urea (CH_4_N_2_O), antibiotic solutions, ethidium bromide (EtBr), acridine orange (AO), Dulbecco’s modified Eagle’s medium (DMEM), and bovine serum albumin were purchased from HiMedia, Mumbai, India. All chemicals employed in this experiment were analytical grade and were utilized without undergoing additional purification. All experiments conducted in this research utilized sterile double distilled water (d/w).

### 2.2. Synthesis of 8-Anilino-1-naphthalenesulfonate-Conjugated Carbon-Coated Manganese Ferrite Nanodots (ANS-MnFe@C)

Using a one-pot hydrothermal synthesis method, we developed manganese ferrite coated with carbon dots (MnFe@C). A 1:2 molar ratio of MnCl_2_·4H_2_O and FeCl_3_·6H_2_O was mixed in 50 mL of distilled water. To this mixture, 0.31 M of C_6_H_8_O_7_ and 0.5 M of CH_4_N_2_O were added and translocated to a Teflon-lined hydrothermal autoclave. The autoclave was airtight and underwent heating at 160 °C for 12 h, followed by a cooling period to reach room temperature (30 ± 3 °C). The reactant mixture was centrifuged at 12,500 rpm for 20 min at room temperature to eliminate the unreacted elements, followed by dispersing the pellet in sterile d/w. The obtained dispersion was collected, the procedure was repeated a couple of times, and the final pellet was collected to dry at 100 °C in a hot air oven. The dried components were then ground into a fine powder to obtain carbon-coated manganese ferrite nanodots (MnFe@C). The MnFe@C stock solution (1 mg/mL) was prepared by dissolving the obtained MnFe@C powder in d/w. Then, 30 µL of variable concentrations of ANS was added to the MnFe@C solution and incubated for 5 min at 30 ± 3 °C to conjugate with the carbon shell existing on the ferrite core and named ANS-MnFe@C.

### 2.3. Sample Characterization

Fourier transform IR spectra of the synthesized MnFe@C and ANS-conjugated MnFe@C were recorded using a Bruker-Alpha ATR/FTIR spectrometer, Billerica, MA, USA. Spectrophotometric characterization was performed by a Shimadzu UV-1800, Kyoto, Japan, and a Jasco spectrofluorimeter (FP-8300), Tokyo, Japan was used to record the excitation and emission spectra. A Malvern Nano-Zs particle size analyzer, Worcestershire, UK was employed to ascertain both the hydrodynamic diameter and surface zeta potential of the particles, utilizing the principle of dynamic light scattering. For vibrating sample magnetometry (VSM) and X-ray diffractometry (XRD), we used a powdered sample. The crystallographic phase of the synthesized nanodots was determined using a Bruker X8 Kappa APEX II sXRD, Karlsruhe, Germany and the magnetism magnitude was calculated using a Lakeshore 7410 VSM, Westerville, OH, USA. An FEI Quanta 200FEG SEM, Houston, TX, USA and JEM-2100 Plus TEM, JEOL Ltd., Tokyo, Japan were used to analyze the size and surface morphology of the particles. Phantom optical imaging and MR imaging were performed at room temperature using an IVIS^®^ Lumina LT, PerkinElmer, Waltham, MA, USA pre-clinical imaging system and Philips Ingenia 3.0 T MRI scanner, Amsterdam, The Netherlands. Fluorescent cellular images were taken using an Olympus fluorescence microscope (BX-51), Gurgaon, India.

### 2.4. Stability Assessment

The stability of the MnFe@C and ANS-MnFe@C was examined using a spectrofluorimeter and dynamic scattering analysis at a different storage temperatures of 4 °C and 30 ± 3 °C (room temperature). The measurements were conducted at 7-day intervals over 35 days to track changes in the emission intensity, hydrodynamic diameter, and surface charge.

### 2.5. Calculation of the Fluorescence Quantum Yield

The fluorescence quantum yield of the synthesized MnFe@C and ANS-MnFe@C was determined by a relative method using Equation (1). An aqueous quinine sulfate solution was used as a reference solution ($\phi_{R}$ = 0.55) to determine the quantum yield of the synthesized nanodots [41,42].

(1) $$\phi_{S}=\phi_{R}\times \frac{Em\;S}{Em\;R}\times \frac{Ex\;R}{Ex\;S}\;$$

where ϕ denotes the fluorescence quantum yield, S denotes the sample, R denotes the reference, Em denotes the emission intensity, and Ex denotes the excitation intensity.

### 2.6. Phantom MRI and Optical Imaging

Increasing concentrations of MnFe@C and ANS-MnFe@C were introduced into water phantoms in 24-well plates at 37 °C. In Philips Ingenia 3.0 T MRI scanner, the phantoms were imaged using T1- and T2-weighted MRI protocols. We employed a T1-weighted FLAIR imaging sequence with the TR set to 3000 ms, TE of 14 ms, FOV measuring 24 × 24, and varying T1 relaxation times within the range of 400–2000 ms to acquire slices with a thickness of 2 mm. The 2 mm thick slices were obtained using a T2-weighted turbo spin-echo sequence with a variable TE = 10–100 ms, TR of 100,000 ms, FOV of 24 × 24, and a 12 ms echo train length. An IVIS fluorescence imaging system was used to conduct phantom imaging. Fluorescent images were obtained using 460 nm-band optical filters and samples taken at increasing concentrations for phantom imaging in 96-well plates.

### 2.7. Methodology of BSA Sensing

To evaluate the selective detection of the bovine serum albumin (BSA) protein, different concentrations of BSA solution (0.015–0.225 mM) were added to 2 mL of ANS, MnFe@C, and ANS-MnFe@C. The stock solution of 15 mM BSA was prepared in aqueous medium to evaluate the selectivity. The measured amount of BSA stock solution was added and mixed thoroughly, and the respective fluorescence emission spectra were recorded at the specific excitation wavelength of 365 nm with a range of 380–700 nm.

### 2.8. In Vitro Biocompatibility Assessment

The in vitro biocompatibility of ANS-MnFe@C was assessed using a human melanoma cell line (A375) and the 3-(4,5-dimethylthiazol-2-yl)-2,5-diphenyltetrazolium bromide (MTT) assay in comparison with unconjugated MnFe@C particles. In 24-well plates, 1 × 10^4^ A375 cells per well were plated in DMEM including 10% FBS and a 1% antibiotic solution at 37 °C, maintaining 5% CO_2_ in the incubator with a humidified atmosphere for 24 h. A standard protocol was followed to perform the MTT assay [43]. Different concentrations of MnFe@C and ANS-MnFe@C (3.6, 11, 18.2, 36, and 53.7 µM) were treated and an additional 24 h incubation period was used. A total of 100 μL of the MTT salt solution at a concentration of 5 mg/mL was introduced into each well and then subjected to 4 h of incubation at 37 °C in a CO_2_ incubator. After the incubation, dimethyl sulfoxide was added to dissolve the formazan crystals, and the OD was measured at 570 nm. The formula given below in Equation (2) was used to calculate the percentage of cell viability according to Harini et al. [43].

% of cell viability = (OD of sample/OD of control) × 100(2)

To assess live and dead cells, a double staining method (AO/EtBr) was used. A mixture of two dyes, 100 µg/mL of AO and 100 µg/mL of EtBr, was added to the cells, incubated at 37 °C for 3 min, and visualized under a fluorescence microscope using appropriate filters.

### 2.9. Drug Loading Studies

Doxorubicin (Dox) was chosen as the model drug, and 1 mL of doxorubicin (1 mM) was incubated in 1 mL of ANS-MnFe@C for 5 min at room temperature. The encapsulation efficiency of doxorubicin loaded with ANS-conjugated MnFe@C (ANS-MnFe@C-Dox) was determined using the formula mentioned below (Equation (3)). The Dox-loaded ANS-MnFe@C was centrifuged for 15 min at 15,000 rpm, and the supernatant of the Dox solution was measured by a UV spectrophotometer at 498 nm.

(3) $$Encapsulation\;efficiency\left(\%\right)=\frac{Total\;Dox-Free\;Dox}{Total\;Dox}\times 100$$

### 2.10. Doxorubicin Release Studies

The drug release pattern of doxorubicin from the synthesized ANS-MnFe@C-Dox in PBS (phosphate-buffered saline) solution at various pHs (4.5, 7.4, and 9.2) was studied using the membrane diffusion method. A total of 2 mL of ANS-MnFe@C-Dox was sealed in a dialysis membrane, and the release of Dox was allowed in 25 mL of PBS solution under stirring conditions. At a regular time interval of 8 h, 2 mL of solution was withdrawn from the beaker, and the optical density was recorded at 498 nm to calculate the release. Fresh PBS (2 mL) was added to the beaker right after each withdrawal to maintain the final volume. This procedure was repeated by replacing the PBS solutions in each medium. The drug release percentages were then plotted for different time intervals to study the release profile of doxorubicin from the formulation.

To determine the drug transport mechanism of Dox from ANS-conjugated MnFe@C, the Korsmeyer–Peppas model was used. When the exact release mechanism of nanoformulations is unclear or when multiple release phenomena are involved, this model is used to explain how the drugs are released from nanoformulations. The experimental data are fitted to the Korsmeyer–Peppas equation, described in Equation (4).

(4) $$\frac{Q_{t}}{Q_{\infty }}={kt}^{n}$$

where *Q*_t_ and *Q*_∞_ are the cumulative drug release at time t and infinite time, respectively. The constant *k* is influenced by the structure and geometric properties of the particles. The variable *t* stands for the release time, and *n* is the diffusion exponent that signifies the drug mechanism. When *n* ≤ 0.34, the release mechanism follows Fickian diffusion (Case I transport), for *n* ≥ 0.85, it is non-Fickian diffusion (case II transport). An intermediate value (0.43 < *n* < 0.85) indicates anomalous transport, combining both processes [44,45].

### 2.11. Anticancer Activity

In vitro, the anticancer activity of doxorubicin-loaded ANS-conjugated MnFe@C (ANS-MnFe@C-Dox) was assessed by the MTT assay in A549 (human lung cancer cells) cells by comparison with free dox. The above-mentioned MTT protocol was followed for cells were treated with different concentrations of Dox and ANS-MnFe@C-Dox (3.6, 11, 18.2, 36, and 53.7 µM), and the OD was recorded at 570 nm.

## 3. Results and Discussion

**Figure 1:**
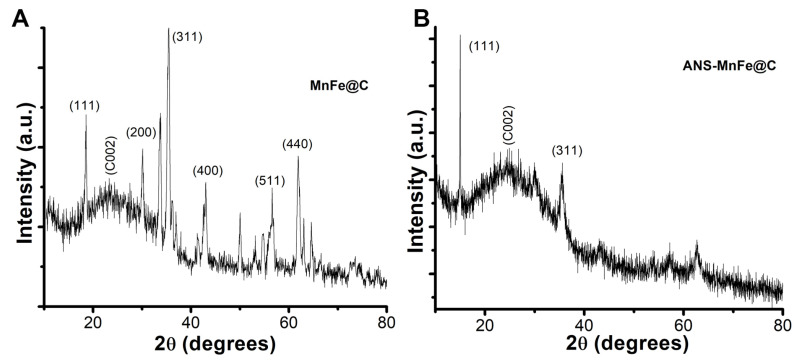
XRD spectral images of MnFe@C (**A**) and ANS-MnFe@C (**B**).

The crystal structure of MnFe@C and ANS-MnFe@C were analyzed by X-ray crystallographic measurements, and the intensity was plotted in the 2θ scale to determine the average size and the crystallinity using Scherrer’s equation (Figure 1). The signature plane (311) of MnFe_2_O_4_ was obtained at a 2θ of 35.43° (Figure 1A). The diffraction pattern supported the formation of the ferrite phase, and the calculated lattice parameter was found to be 8.4375 Å, which was in agreement with the standard JCPD value (JCPDS card #074-2403). The calculated crystalline diameter was 31.84 nm. The reflection peak obtained at 23.14° indicates a turbostatic phase of carbon representing an imperfect graphite crystal or phase. Figure 1B presents the X-ray diffractogram of ANS-MnFe@C, which was a bit noisy and broad compared to MnFe@C, indicating an amorphous nature.

**Figure 2:**
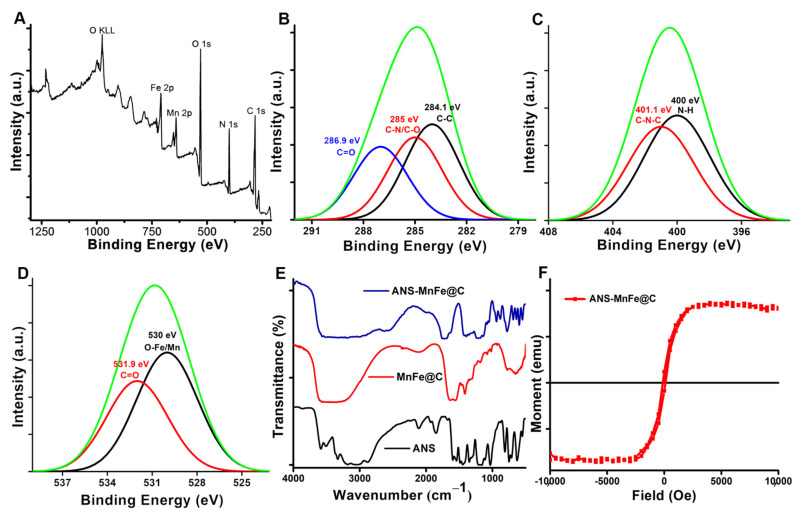
(**A**) XPS analysis of MnFe@C. (**B**–**D**) Deconvoluted XPS spectra of C 1s, N 1s, and O 1s, respectively. (**E**) FTIR spectra of ANS, MnFe@C, and ANS-MnFe@C. (**F**) Room temperature magnetic hysteresis loop of ANS-MnFe@C.

The surface chemistry of MnFe@C was further examined using X-ray photoelectron spectroscopy (XPS) to determine the elemental composition and the chemical states (Figure 2A). Scanning was performed over a binding energy range of 1250 to 250 electron volts (eV). The XPS survey spectrum revealed the presence of Fe 2p (711.1 eV), Mn 2p (642.6 eV), O 1s (533.6 eV), N 1s (401.3 eV), and C 1s (284.2 eV) at their corresponding binding energy levels. A couple of peaks were observed at higher binding energy zones that can be assigned to the release of Auger electrons and represented as ‘O KLL.’ The obtained XPS data were consistent with the standard data representing carbon-coated manganese ferrite synthesis. The deconvoluted C 1s spectra (Figure 2B) showed three different types of carbon bonds: C=O (286.9 eV), C-N/ C-O (285 eV), and C-C (284.1 eV). Likewise, the peaks at 401.1 eV and 400 eV were assigned to C-N-C and N-H, respectively, in Figure 2C. The O 1s spectrum was deconvoluted to two peaks (Figure 2D) of 531.9 eV for C=O and at 530 eV for O-metal (Fe/Mn).

The chemical composition and surface functional groups of ANS-MnFe@C were studied by FTIR (Figure 2E). The FTIR spectra of ANS, MnFe@C, and ANS-MnFe@C were recorded in the range of 500 cm^−1^ to 4000 cm^−1^ using KBr as a reference. The formation of metal oxide was confirmed by observing the Fe-O stretching band at 551 cm^−1^. The C-N stretching band was observed at 1145 cm^−1^ and 1234 cm^−1^. Bands at 1428 cm^−1^ and 1632 cm^−1^ were due to the symmetric and asymmetric bending vibration of C=O. The large broad band at 3564 cm^−1^ to 3277 cm^−1^ was attributed to the O-H stretching vibration of OH^−^ groups. The recorded FTIR bands support the decoration of the MnFe@C nanodots. C-H stretching of a strong vibration peak of the aromatic ring appeared at 3016 cm^−1^. The peak at the 1362 cm^−1^ band showed O-H plane bending. Symmetric and asymmetric stretching of SO_3_ groups showed a tensile vibration peak at 1053 cm^−1^ and 1143 cm^−1^. The bending vibration of the O-H bond was seen at 882 cm^−1^. These significant FTIR bands confirmed the conjugation of 8-anilino-1-naphthalenesulfonic acid on MnFe@C. The magnetic behavior of the ANS-conjugated nanodots was measured by VSM, shown in Figure 2F. The VSM result did not show any apparent hysteresis loop, indicating that the synthesized magnetic particles possess superparamagnetic behavior at room temperature, making them highly useful for MRI as contrast agents. The saturation magnetization (Ms) values of ANS-MnFe@C nanodots were 33.59 emu. This indicates that the optimized hydrothermal method can produce highly crystalline and phase-pure nanoparticles with a superparamagnetic nature.

### 3.1. Optical Properties

**Figure 3:**
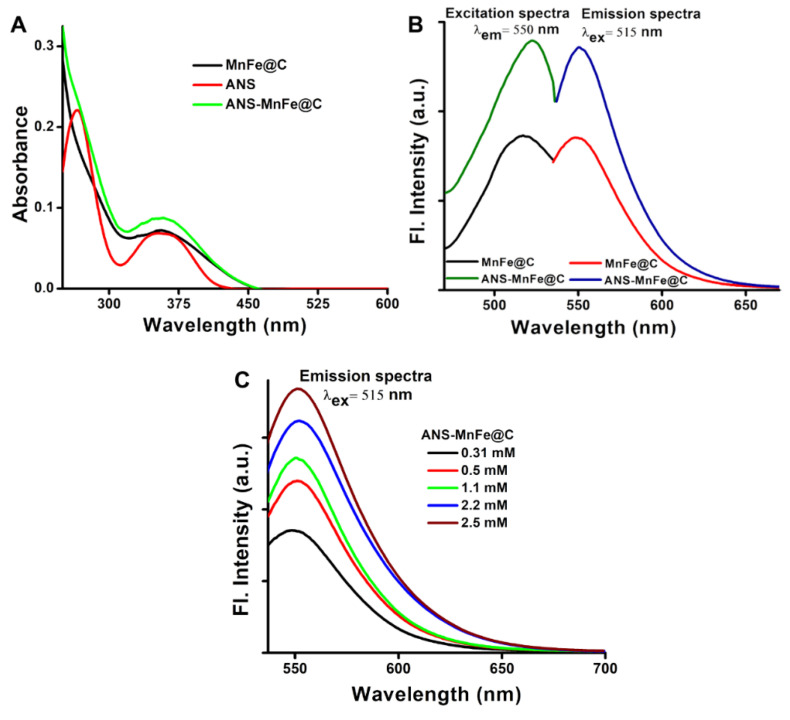
(**A**) Absorption spectra of MnFe@C, ANS, and ANS-MnFe@C. (**B**) Fluorescence spectra [emission (λ_ex_ = 515 nm)/excitation (λ_em_ = 550 nm)] of MnFe@C and ANS-MnFe@C. (**C**) Emission spectra of ANS-MnFe@C containing increasing ANS concentrations.

UV-Vis absorption and steady-state fluorescence spectroscopy were used to analyze the optical behaviors of MnFe@C and ANS-MnFe@C. The absorption spectra of synthesized MnFe@C nanodots have a broad absorption peak ranging from 250 to 300 nm, and a narrow peak at 358 nm is attributed to the formation of CDs. The ANS shows two distinct peaks at 355 nm and 265 nm as a signature. The ANS-conjugated MnFe@C shows similar absorption spectra with higher absorption at 355 nm that is more stretched than the non-conjugated MnFe@C nanodots, as shown in Figure 3A. It is firmly established that neither urea nor citric acid has been shown to be capable of absorbing light at the given wavelength. The conjugation of ANS could have resulted in the change in absorbance due to changes in its microenvironment around the MnFe@C.

The steady-state fluorescence spectra of the MnFe@C and ANS-MnFe@C are shown in Figure 3B. The synthesized particles showed an intriguing emission behavior. When excited at 515 nm, MnFe@C showed a maximum emission intensity (λ_em_) at 550 nm. Upon excitation of the synthesized MnFe@C at its maximum wavelength (λ_max_ = 515 nm), a weak emission peak was observed at 550 nm with an intensity of 852 a.u. In contrast, the ANS-conjugated MnFe@C exhibited a more pronounced emission peak at 550 nm with a significantly higher intensity of 1358 a.u. when excited at the same wavelength (515 nm) and at the same concentration. This was further validated by excitation spectra, where the emission wavelength was fixed at 550 nm, yielding a fluorescence peak at 515 nm (Figure 3B). The effects of ANS conjugation were examined using emission spectra (Figure 3C), which showed a significant increase in fluorescence emission for ANS-conjugated MnFe@C compared to MnFe@C dots when excited at the absorption zone of MnFe@C (515 nm) where ANS does not have any absorption. This conjugation was attributed to the electrostatic interaction between amine groups existing on the surface of carbon dots and the sulfonate groups in ANS. The fluorescence emission of the carbon dots (CDs) remained independent of the excitation wavelength due to the abundant amine functional groups present on their surface.

### 3.2. Investigation of the Quantum Yield

The relative fluorescence quantum yield for MnFe@C and ANS-MnFe@C was determined using quinine sulfate in 0.1 M H_2_SO_4_ as a standard reference, and the quantum yield was measured to be 56% and 72%, respectively. Due to the abundant surface functional groups, MnFe@C can passivate surface defects, reducing non-radiative recombinant pathways and increasing the probability of radiative recombination to enhance the quantum yield. The conjugation of ANS can lead to better surface passivation of MnFe@C by forming stable complexes or modifying surface functional groups. This enhanced surface passivation can reduce non-radiative recombination pathways, leading to an increased quantum yield.

### 3.3. Bandgap Energy Measurement

A material’s optical properties arise from its interaction with electromagnetic radiation, involving processes such as absorption, emission, transmission, reflection, refraction, scattering, and diffraction. In semiconductors, these properties, which are usually observed in the UV-visible or infrared spectra, are closely related to their electronic band structure and atomic makeup [46]. Examining the optical absorption edges of semiconductors helps us understand the energy gap between the valence and conduction bands, known as the bandgap, which is crucial when evaluating their potential for device applications. The band gap can be determined using techniques such as electrical and photoconductivity, the Hall effect, or optical absorption. Measuring bandgap energy is crucial for understanding the optical properties of semiconductors. The Tauc plot is essential for determining the band gap of semiconductor materials, which plays a key role in biosensing, stimuli-responsive drug delivery, bioimaging, and photodynamic therapy (PDT). In PDT, materials with precisely controlled band gaps are valuable because they enable the activation of nano-enabled photosensitizers. Today, semiconductors are frequently used in drug delivery. Tauc plots are instrumental in identifying the energy needed to understand how specific wavelengths of light interact with semiconductor-based drug delivery systems. Quantum dots and carbon dots, with their adjustable band gaps, hold great potential for bioimaging and theranostics, and Tauc plots are crucial for tuning their absorption energy within the electromagnetic spectrum. The bandgap energies of the MnFe@C and ANS-MnFe@C were evaluated by the Tauc method using optical absorption spectra based on the empirical formula expressing the energy-dependent absorption coefficient as follows [47,48]:

(αhυ)^γ^ = A (hυ − E_g_) (5)

Here, α is the absorption coefficient, υ is the frequency of light, h is Planck’s constant, E_g_ is the bandgap energy, and A is a proportionality constant. The exponent γ depends on the nature of the electronic transition causing the absorption; γ = 2 for direct transitions, and γ = 1/2 for indirect transitions. Representing Equation (5) for an indirect allowed transition, where γ equals 1/2 and assuming the transition probability is unity, the calculation can be performed as follows [49,50]:

(αhυ)^1/2^ = A(hυ − E_g_) (6)

**Figure 4:**
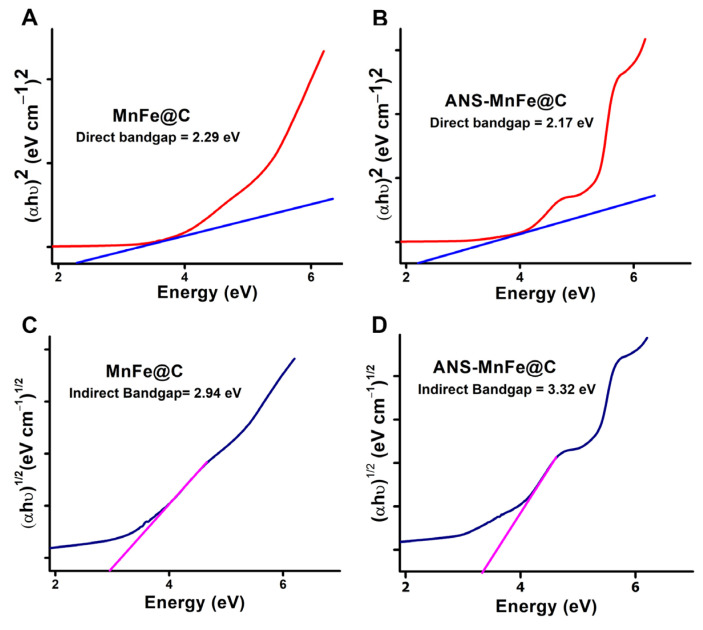
Band gap energy estimations using the Tauc plot. Direct bandgaps of (**A**) MnFe@C and (**B**) ANS-MnFe@C, and indirect bandgaps of (**C**) MnFe@C and (**D**) ANS-MnFe@C.

The extrapolation of (αhυ)^2^ (eVcm^−1^)^2^ and (αhυ)^1/2^ (eVcm^−1^)^1/2^ vs. energy (eV) graphs (Figure 4) determined the optical bandgap energy of MnFe@C and ANS-MnFe@C. The linear and nonlinear part of the curves showed the allowed transition characteristics. The Tauc method relies on a traced tangent line just before the linear region of the curve. For direct allowed electron transitions (Figure 4A,B) and indirect allowed electron transitions (Figure 4C,D), the intercept of the extrapolated linear part of the plot (αhυ)^γ^ against hυ with abscissa helped to determine the E_g_ value.

The bandgaps for the MnFe@C and ANS-MnFe@C were calculated as 2.94 eV and 3.32 eV using the curve on the indirect bandgap by a linear fit. The indirect bandgap energies of MnFe@C and ANS-MnFe@C are matching with the excitation and emission values. The extended (π) conjugation of ANS increases the energy required for electron excitation, resulting in a higher bandgap energy compared to non-conjugated MnFe@C. The changes in the bandgap energy measurement of the ANS-conjugated MnFe@C make them highly capable wide bandgap semiconductor materials with extensive voltaic features of sp2 carbon to provide a pathway for enhanced efficient, lighter, and greater tolerance to high temperature.

### 3.4. Particle Size Analysis

**Figure 5:**
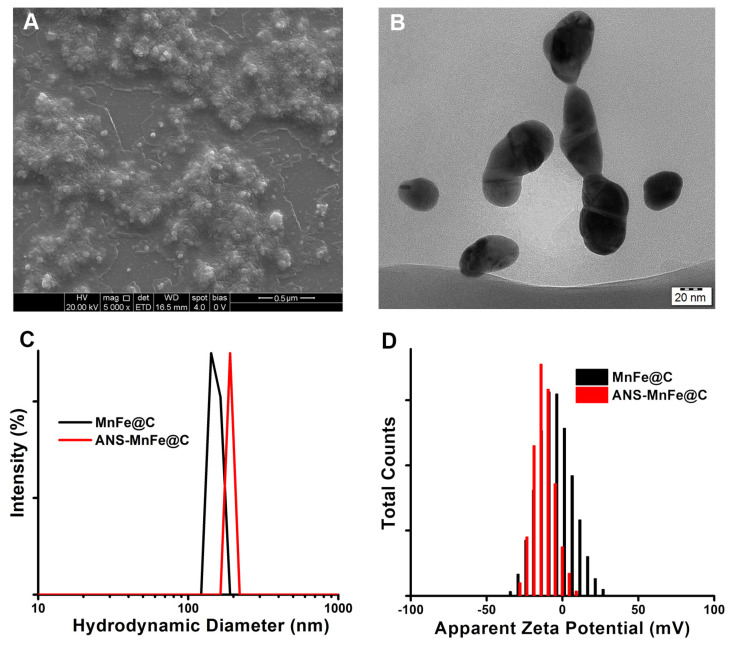
(**A**) SEM and (**B**) TEM images of ANS-MnFe@C; (**C**) DLS and (**D**) seta potential studies of MnFe@C and ANS-MnFe@C.

The crystal structures and surface morphology of the nanodots were investigated by TEM and SEM analyses. The SEM image of ANS-MnFe@C is shown in Figure 5A, which shows that the nanodots have uniform cubic structures. TEM images (Figure 5B) of ANS-MnFe@C nanodots were taken to better understand the nanostructure of synthesized materials. They show the crystal structure of the ANS-MnFe@C, with an average diameter of 33 ± 3 nm. The MnFe core is denoted by a central black spot, while the surrounding shadow signifies the presence of a carbon shell. To determine the hydrodynamic diameter (dH) and the polydispersity index (PDI), we carried out DLS measurements. The PDI indicates the degree of dispersion of nanoparticles in a colloidal solution, and the hydrodynamic diameter (dH) indicates the size of the nanoparticles associated with the hydration layer surrounding the nanoparticles. As shown in Figure 5C, the dH of the MnFe@C was 142 nm, whereas the dH of ANS-MnFe@C was 190.1 nm with a PDI of 0.14. The DLS measurement showed that the PDI values were within the limit, and synthesized particles were monodispersed [51]. In colloidal suspensions, the zeta potential is used to assess nanoparticle stability and the surface-coating quality at the edge of their double layer. As seen in Figure 5D, the zeta potentials of MnFe@C and ANS-MnFe@C were −8.15 mV and −14.9 mV. After conjugating ANS to the surface of carbon dots, the lower negative value of the nanodots increased from −8.15 mV for MnFe@C to −14.9 mV for ANS-MnFe@C, demonstrating the successful conjugation of ANS on the surface of carbon dots and the enhanced stability. ANS-conjugated nanodots were well dispersed in an aqueous solution as a result of their negative surface charge value as well as accompanying electrostatic repulsion. The higher surface charge of ANS-MnFe@C indicated that the conjugation improved the stability of the nanodots.

### 3.5. Stability Studies

**Figure 6:**
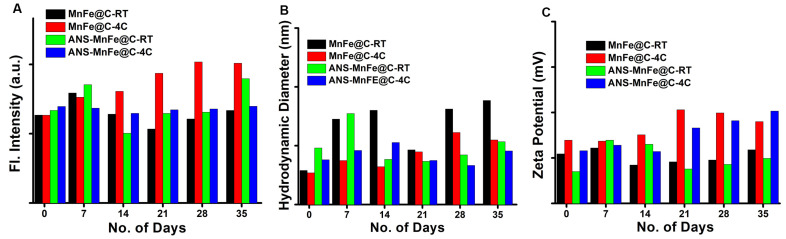
Stability of the MnFe@C and ANS-MnFe@C are different storage temperatures using (**A**) the emission intensity, (**B**) hydrodynamic diameter, and (**C**) zeta potential.

The stability of the MnFe@C and ANS-MnFe@C was studied for a period of 35 days, while the emission intensity (Figure 6A), size (Figure 6B), and surface charge (Figure 6C) were recorded on every seventh day of storage. The samples stored at room temperature exhibited variations in emission intensity, size, and surface charge, whereas those stored at 4 °C showed significantly fewer changes. This indicates that samples kept at 4 °C are more stable and suitable for long-term use.

### 3.6. Phantom MRI

**Figure 7:**
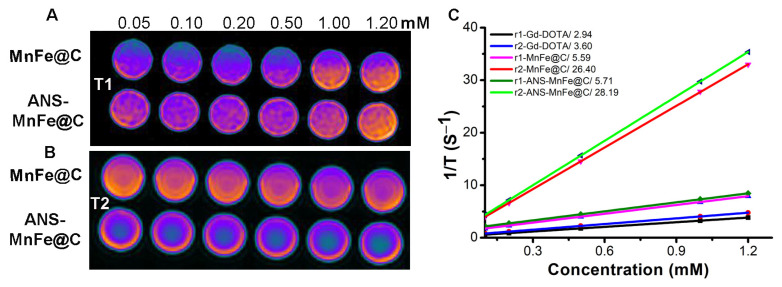
(**A**) T1-weighted and (**B**) T2-weighted phantom images of MnFe@C and ANS-MnFe@C with increasing concentrations. (**C**) The calculated relaxivity was plotted against the concentration and compared with commercially available Gd-DOTA.

To test the applicability of these nanodots as MRI contrast agents, an in vitro MR imaging study was carried out for MnFe@C and ANS-MnFe@C. Figure 7A presents T1-weighted phantom MR images of the nanodots with variable concentrations. Both conjugated and unconjugated MnFe@C were utilized in 24-well plates to observe phantom MRI conducted on a 3 T scanner. Using the DICOM image processing program and ImageJ software, intensities were measured to calculate the relaxivity. Due to the presence of manganese in nanodots, the synthesized MnFe@C showed a positive contrast effect. From the MRI images, we observed that the signal intensity change was proportional to the concentration. A corresponding result was observed for the ANS-conjugated MnFe@C nanodots. The molar longitudinal relaxivity (r1) of MnFe@C was found to be 5.59 mM^−1^s^−1^, and that of ANS-MnFe@C was 5.71 mM^−1^s^−1^ (Figure 7C). In the T2-weighted MR images, the presence of ferrite in the structure of MnFe@C showed a negative contrast effect, as shown in Figure 7B. The MnFe@C nanodots showed a moderate change compared to the ANS-conjugated ones, but with the concentration changes, there was not much variation in the intensities. The molar transverse (r2) relaxivity was found to be 26.40 mM^−1^s^−1^ and 28.19 mM^−1^s^−1^, respectively, for MnFe@C and ANS-MnFe@C. Similar concentrations of the commercially used contrast agent Gd-DOTA were used to compare the relaxivity efficacy, and it was found that the Gd-DOTA showed an r1 and r2 relaxivity of 2.94 and 3.60 mM^−1^s^−1^, respectively, which were much lower than the engineered nanodots (Figure 7C). The r2/r1 ratio was calculated for MnFe@C and ANS-MnFe@C, and it was 4.71 and 4.94, respectively, belonging in the range of 3 to 10, indicating the capacity of being a dual contrast agent (T1 as well as T2) for MRI [52,53].

### 3.7. Phantom Optical Imaging

**Figure 8:**
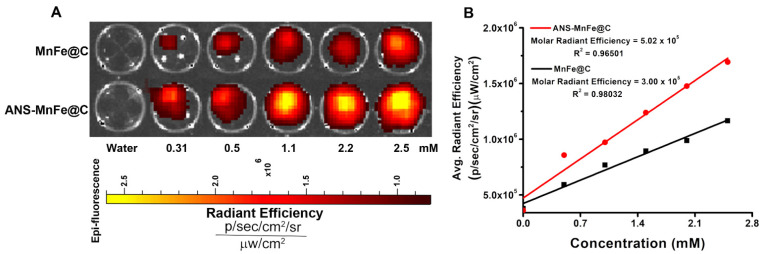
(**A**) Phantom optical images of MnFe@C and ANS-MnFe@C with different concentrations. (**B**) The molar radiant efficiencies were determined by plotting fluorescence image intensities against the concentration.

Figure 8A illustrates the optical properties of MnFe@C and ANS-MnFe@C as fluorescence imaging probes in the small-animal imaging system. In this study, we found that increasing concentrations of nanodots increased the fluorescence intensities; all fluorescent intensities were normalized to photons/second/centimeter^2^/steradian (p/s/cm^2^/sr), and the background intensities were subtracted. Increasing concentrations of ANS-conjugated and unconjugated nanodots showed a significant increase in intensities. With respect to the concentration of nanodots, the average radiant efficiencies are shown in the graph (Figure 8B), where the slope of MnFe@C and ANS-MnFe@C were determined as 3.00 × 10^5^ and 5.02 × 10^5^ p/s/cm^2^/sr per mM, corresponding to the average radiant efficiencies. The results indicated that the particles conjugated with ANS exhibited superior radiant efficiencies in comparison to unconjugated particles. It was observed that the obtained results correlated closely with the steady-state fluorescence results.

### 3.8. Protein Sensing Properties of ANS-MnFe@C

**Figure 9:**
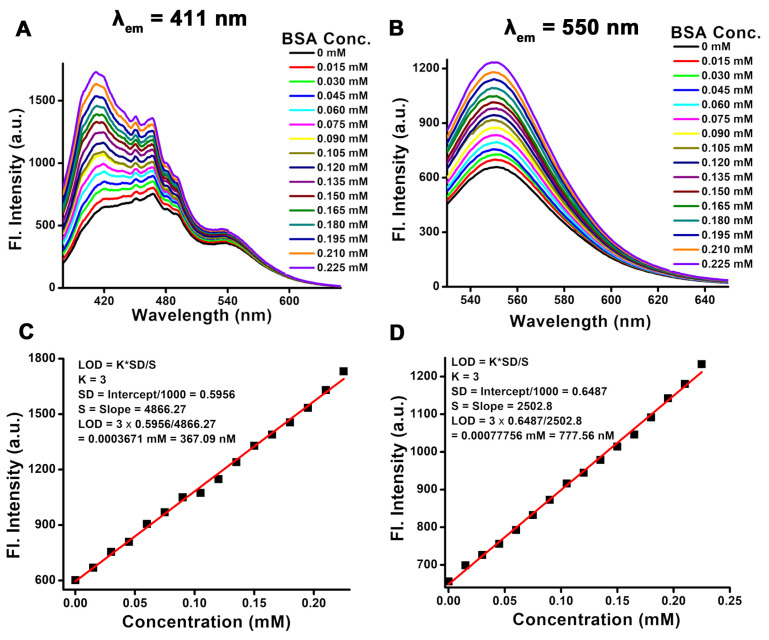
Fluorescence emission spectra of ANS-MnFe@C show the increasing intensity upon the addition of an increasing amount of the BSA protein. (**A**) λ_ex_ = 365 nm and λ_em_ = 411 nm, (**B**) λ_ex_ = 515 nm and λ_em_ = 550 nm. (**C**,**D**) The fluorescence intensity was plotted against the concentration for λ_em_ = 411 nm and λ_em_ = 550 nm, respectively.

To evaluate the sensing capabilities of ANS-MnFe@C, various concentrations of BSA were introduced, and the corresponding fluorescence intensities were measured. The steady-state fluorescence behavior was examined by focusing on ANS (λ_ex_ = 365 nm) and carbon dots (λ_ex_ = 515 nm) present in ANS-MnFe@C, as both exhibit fluorescence in distinct regions. Figure 9A demonstrates the increase in fluorescence at λ_em_ = 411 nm as BSA was gradually added. The fluorescence intensity of ANS showed a consistent increase with BSA concentrations ranging from 0.015 to 0.225 mM (Figure 9C), attributed to ANS binding to the hydrophobic regions of the protein. A linear response was observed within this concentration range, with a detection limit (LOD) of 367 ± 71 nM and a quantification limit (LOQ) of 1224 ± 62 nM. A similar trend was observed at an emission wavelength of 550 nm when ANS-MnFe@C was excited at 515 nm (Figure 9B,D). Here, the LOD was 777.56 ± 35 nM and the LOQ was 2591.89 ± 27 nM, nearly twice that of ANS. The amine (NH_2_) and hydroxyl (OH) groups in MnFe@C are capable of forming strong interactions with BSA via hydrogen bonding, contributing to the sensing mechanism. This study confirms that ANS is more accessible to protein molecules compared to the carbon shell surrounding the magnetic core, owing to MnFe@C’s functionalization with ANS. Additionally, the presence of anilinosulfonic acid groups on MnFe@C enhances the sensitivity of BSA detection. The sulfonic acid groups offer extra binding sites for BSA through electrostatic interactions, while the aromatic ring of aniline engages in π-π interactions with BSA’s aromatic residues, further improving sensitivity.

### 3.9. Binding Efficiency and Stability Constants

**Figure 10:**
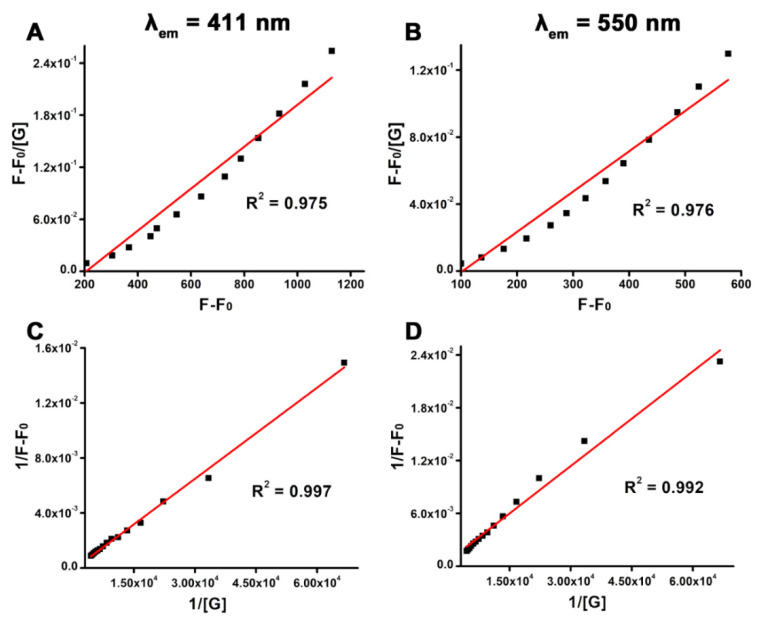
(**A**,**B**) Scatchard plots for ANS-MnFe@C and BSA binding monitored at λ_em_ = 411 and 550 nm, respectively. (**C**,**D**) Benesi–Hildebrand (BH) plots of ANS-MnFe@C in the presence of BSA monitored at λ_em_ = 411 and 550 nm, respectively.

The adjusted Scatchard (Equation (7)) and Benesi–Hildebrand (Equation (8)) equations were applied to find the stability or association constant (K_a_). Figure 10A,B shows the Scatchard plot for BSA binding to functionalized ANS and carbon dots on ANS-MnFe@C [54,55,56].

(7) $$\left(F-F_{0})/[G]=K_{a}(F_{\infty }-F_{0}\right)-K_{a}(F-F_{0})$$

(8) $$1/(F-F_{0})=1/\left(F-F_{0}\right)K_{a}\left[G\right]+1/(F-F_{0})$$

where F_0_ = the fluorescence intensity in the absence of BSA, F = the fluorescence intensity at the working concentrations, F_∞_ = the fluorescence intensity at maximum concentration, and [G] represents the concentrations of proteins. Figure 10A, B depicts the binding of BSA to ANS and to carbon dots present in ANS-MnFe@C analyzed using the Scatchard equation. The X-axis represents (F − F_0_), while the Y-axis corresponds to (F − F_0_/[G]). The modified Scatchard equation derived from Figure 10A yielded an association constant (K_a_) of 4988 M^−1^, with an R^2^ of 0.975. Similarly, Figure 10B produced a K_a_ of 2471 M^−1^, with an R^2^ of 0.976. Furthermore, the adjusted B-H plot (Figure 10C) resulted in a K_a_ of 4530 M^−1^, with an R^2^ of 0.997, while Figure 10D provided a K_a_ of 2790 M^−1^, with an R^2^ of 0.992. The association or stability constants derived from both the Scatchard and B-H plots showed strong agreement.

### 3.10. In Vitro Biocompatibility Assessment

**Figure 11:**
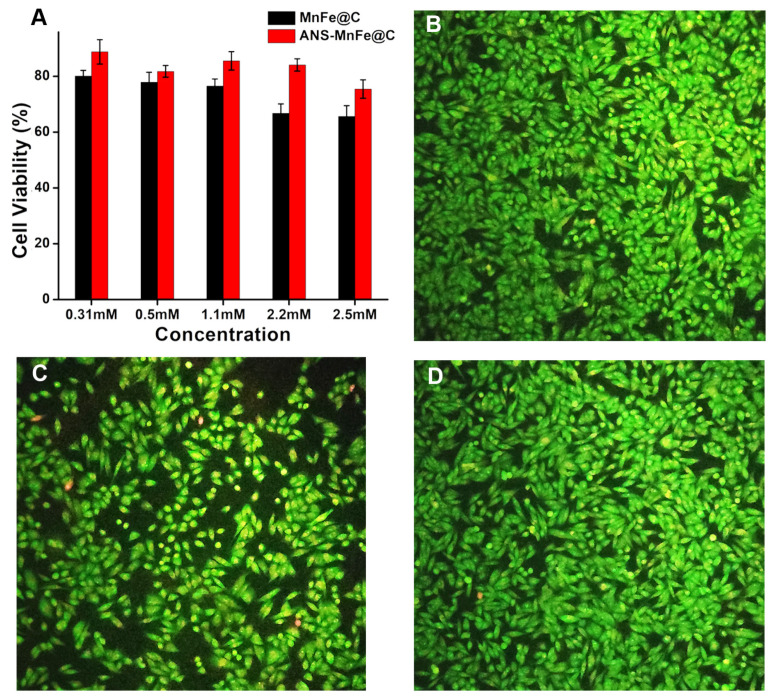
(**A**) Viability of A375 cells treated with different concentrations (0.31, 0.5, 1.1, 2.2, and 2.5 mM) of ANS-conjugated and unconjugated MnFe@C; fluorescence images of AO/EtBr-stained (10× magnification) A375 cells: (**B**) control; (**C**) MnFe@C; and (**D**) ANS-MnFe@C.

The biocompatibility of the synthesized ANS conjugated and unconjugated MnFe@C was evaluated using an MTT assay in the A375 cell line (human melanoma cell line). MTT is converted to formazan in the presence of mitochondrial succinate dehydrogenase, a critical enzyme involved in the TCA cycle and a part of the respiratory enzyme complex. The assay was performed using the standard protocol, and the background absorbance of the solvent in which the nanostructure was dissolved was subtracted from the sample values. Performing this, we could clearly mention that the engineered nanostructure was benign. Based on the outcome of this study, it was determined that cells treated with the MnFe@C conjugated with ANS demonstrated greater viability in comparison with those treated with unconjugated MnFe@C (Figure 11A). A live/dead assay was performed by a double staining procedure using acridine orange/ethidium bromide, in which dead cells are stained red and live cells are stained green. Figure 11B–D shows the control cells, 2.2 mM MnFe@C-, and 2.2 mM ANS-MnFe@C-treated cells. In the control group, all the cells are alive, while in the treated cells, 66.78% and 84.1% cell viability are shown. This result shows that the viability rate of cells treated with ANS-conjugated MnFe@Cs is better compared to cells treated with the unconjugated MnFe@C. The ANS conjugation on the surface of nanodots drastically reduced the toxicity of manganese present in the MnFe@C; it shows the biocompatibility and negligible toxicity of synthesized ANS-MnFe@C.

### 3.11. Drug Loading, Release Kinetics, and Anticancer Assessment

**Figure 12:**
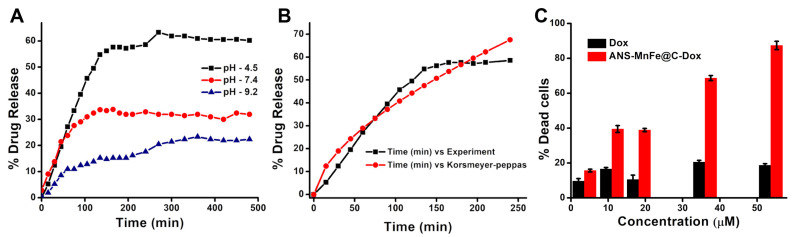
(**A**) Drug release profile for-Dox loaded ANS-MnFe@C in different release media (pH 4.5, 7.5, and 9.2). (**B**) The Korsmeyer–Peppas model was used to profile Dox release from ANS-MnFe@C. (**C**) Percentage of A549 cells killed after treatment with Dox and ANS-MnFe@C-Dox.

The drug loading efficiency of ANS-MnFe@C-Dox was found to be 68.6%, and by recoding, the OD of Dox was monitored at 498 nm. The release profile of Dox from ANS-MnFe@C-Dox is shown in Figure 12A, and it was determined in three different release media (PBS buffer, pH 4.5, 7.4, and 9.2) for 480 min. Burst release was observed for the first few hours due to the unloaded drug molecules (dox) on the surface of ANS-MnFe@C-Dox. Subsequently, sustained release of Dox occurred. After 480 min, 23% release was observed in the basic medium (pH 9.2), and 33.6% release was observed in the neutral medium (pH 7.4), but in the acidic environment (pH 4.5), 63.3% drug release was observed. The release kinetics at different pHs within 480 min clearly indicated that pH strongly influences Dox release from ANS-MnFe@C-Dox (Figure 12A). The results may be due to the fact that the sulfonate groups on the ANS-MnFe@C can become protonated in an acidic environment. This protonation reduces the electrostatic interactions between the dox and ANS-MnFe@C, leading to faster release of the drug. Also, dox is more soluble under acidic conditions, which can enhance its release from the ANS-MnFe@C. These mechanisms make ANS-MnFe@C particularly effective for targeted drug delivery under acidic conditions, such as cancerous tissues.

The drug release mechanism from the nanoparticles was identified by the Korsmeyer–Peppas model. The acidic medium release (pH 4.5) data were used to plot the graph of Q_t_/Q_∞_ vs. time, as shown in Figure 12B. The r, k, and *n* values found from the Korsmeyer–Peppas model were 0.97, 2.37, and 0.61. The *n* value of pH 4.5 data indicates an anomalous transport mechanism involved in drug release when the *n* value is in the 0.43 < *n* < 0.85 range. This anomalous transport mechanism shows that drug release from the ANS-MnFe@C involves deviations from classical Fickian diffusion, where the release rate is influenced by a combination of factors.

In vitro, anticancer activity was assessed using A549 cells to determine the percentage of dead cells after treatment with Dox and ANS-MnFe@C-Dox, as shown in Figure 12C. The cell killing efficiency of higher concentrations (53.7 µM) of ANS-MnFe@C-Dox was found to be 87% and 19% for free Dox. The cell-killing efficacy of ANS-MnFe@C-Dox was three times higher than Dox alone. Due to the smaller size and surface properties, ANS-MnFe@C-Dox facilitates better cellular uptake and protects dox from degradation in the biological environment.

## 4. Conclusions

In conclusion, a hydrothermal method was utilized to synthesize carbon-coated manganese ferrite (MnFe@C) nanodots, which were then linked with 8-anilino-1-naphthalenesulfonic acid (ANS) to enhance their fluorescence and magnetic resonance imaging capabilities, as well as improve their biocompatibility while reducing toxicity. Each stage of particle development was analyzed using various techniques to verify the composition, structure, and physicochemical properties. The TEM images clearly reveal that the magnetic core is coated with a carbon shell approximately 33 ± 3 nm thick, and in vitro, MR imaging studies demonstrate that the engineered nanodots can capture both T1- and T2-weighted images. The carbon shell also endowed the particles with fluorescent properties, enabling them to be used for fluorescence-based imaging. Surface modification of the nanodots with ANS improved their accuracy in fluorescence imaging. ANS-MnFe@C demonstrated a higher quantum yield of 72% and enhanced protein sensitivity, with the binding efficiency reflected by changes in fluorescence intensity that were attributed to both the ANS and the carbon shells present on ANS-MnFe@C. This was further confirmed through Scatchard and Benesi–Hildebrand plots. The addition of the fluorescent probe ANS enhanced the biocompatibility and bioavailability of the particles, as demonstrated by in vitro studies with cell lines. Furthermore, the synthesized particles displayed minimal toxicity risks at high concentrations when compared to the unconjugated particles, and Dox loading made them a potential drug delivery system for treating cancer cells. Overall, the engineered ANS-MnFe@C nanodots exhibit superior sensitivity, biocompatibility, bioavailability, and a low cost, making them promising candidates for use as theranostic agents in multimodal imaging and protein sensing applications.

## Data Availability

All the data related to this study have been incorporated in the article.
